# Editorial: Closed-Loop Systems for Next-Generation Neuroprostheses

**DOI:** 10.3389/fnins.2018.00026

**Published:** 2018-02-12

**Authors:** Timothée Levi, Paolo Bonifazi, Paolo Massobrio, Michela Chiappalone

**Affiliations:** ^1^Laboratoire IMS, UMR Centre National de la Recherche Scientifique 5218, University of Bordeaux, Bordeaux, France; ^2^Biocruces Health Research Institute, Bilbao, Spain; ^3^School of Physics and Astronomy, Tel Aviv University, Tel Aviv, Israel; ^4^Department of Informatics, Bioengineering, Robotics and System Engineering, University of Genova, Genova, Italy; ^5^Rehab Technologies, Istituto Italiano di Tecnologia, Genova, Italy

**Keywords:** neuroprostheses, neuromodulation, stimulation, closed-loop experiments, neuronal circuits, artificial spiking neural network

Millions of people worldwide are affected by neurological disorders which disrupt the connections within the brain and between brain and body causing impairments of primary functions and paralysis. Such a number is likely to increase in the next years and current assistive technology is yet limited. A possible response to such disabilities, offered by the neuroscience community, is given by Brain-Machine Interfaces (BMIs) and neuroprosthetic research.

The latter field of research is highly multidisciplinary, since it involves very different and disperse scientific communities, making it fundamental to create connections and to join research efforts. Indeed, the design and development of neuroprostheses involve different research topics such as: interfacing to nervous systems at different levels of architectural complexity (from *in vitro* neuronal ensembles to human brain), bio-electronic interfaces for stimulation (e.g., micro-stimulation, DBS: Deep Brain Stimulation) and recording (e.g., EMG, Electromyography; EEG, Electroencephalography; LFP, Local Field Potential), innovative signal processing tools for coding and decoding of neural activity, biomimetic artificial Spiking Neural Networks (SNN) and neural network modeling (Indiveri et al., 2001; Bonifazi et al., 2013). In order to develop functional communication with the nervous system and to create a new generation of neuroprostheses, the study of closed-loop systems is mandatory. It has been widely recognized that closed-loop neuroprosthetic systems achieve more favorable outcomes than open-loop devices. Improvements in task performance, usability, and embodiment have all been reported in systems utilizing some form of feedback. The bi-directional communication between living neurons and artificial devices is the main final goal of those studies. However, closed-loop systems not only based on visual feedback are still uncommon, mostly due to requirement of multidisciplinary effort. Only few examples in this direction can be cited from the literature, such as O'Doherty et al. (2011) and Capogrosso et al. (2016). Therefore, through this research topic on closed-loop systems for next-generation neuroprostheses, we encourage an active discussion among neurobiologists, electrophysiologists, bioengineers, computational neuroscientists, and neuromorphic engineers.

This Editorial aims to facilitate this process by ordering the 25 contributions of this research in which we highlighted in three different parts: (A) Optimization of different blocks composing the closed-loop system, (B) Systems for neuromodulation based on DBS, EMG, and SNN, and (C) Closed-loop BMIs for rehabilitation.

## (A) optimizing the different blocks composing a closed-loop system

To design closed-loop neuroprostheses, the three main blocks which require optimization are recording, signal processing, and stimulation.

To target specific structures with micro-stimulation, localization methods should be developed. For that, Telkes et al. focuses on multiple spectral features extracted from microelectrode-recorded LFPs which could be used to automate the identification of the optimal track and the SubThalamic Nucleus (STN) localization. These results establish the initial evidence that LFPs can be strategically fused with computational intelligence in the operating room for STN localization and the selection of the track for chronic Deep Brain Stimulation (DBS) electrode implantation.

After recording electrical activities of the brain or specific neural network, the coding part follows. For example a decoder translates recorded neural activity into motor commands while an encoder delivers sensory information collected from the environment directly to the brain creating a closed-loop system. Yang et al. define a novel decoding algorithm regardless of the number of neurons generating the recorded signals. Gailey et al. describe a proof of concept for online EMG-based decoding of hand postures and Individual digit forces for prosthetic hand control. Courellis et al. propose an algorithmic and computational framework for identifying cortical networks across subjects in which dynamic causal connectivity is modeled among user-selected cortical regions of interest. Li et al. aim to improve accuracy of signal processing by designing a better encoding model of primary motor cortical activity during hand movements and combining this with decoder engineering refinements, resulting in a new unscented Kalman filter-based decoder.

Another important element to consider in closed-loop systems is the use of artificial SNNs to replace lost neural networks, or to implement artificial intelligence in the signal processing decoder. Pani et al. present a modular and efficient FPGA design of an *in silico* SNN exploiting the Izhikevich model. The proposed system is able to simulate a fully connected network counting up to 1,440 neurons, in real-time, at a sampling rate of 10 kHz, which is reasonable for small to medium scale extra-cellular closed-loop experiments. Boi et al. create a bidirectional BMI which establish a two-way direct communication link between the brain and the external world. As a first step toward this goal, they developed a modular bidirectional BMI setup that uses a compact neuromorphic processor as a decoder. On this chip, a network of SNNs is built using its ultra-low-power mixed-signal analog/digital circuits. Kohno et al. review different SNNs and propose qualitative neuron models for designing more biomimetic SNN.

For BMI system, optimization of Movement Related Cortical Potential (MRCP) recordings is important to improve control of a neural prosthesis. MRCP, a slow cortical potential from the scalp EEG, has been used in real-time brain-computer-interface (BCI) systems designed for neurorehabilitation. Karimi et al. propose a new MRCP detection method based on constrained independent component analysis (cICA). Xu et al. investigate single trial EEG traces during motor imagery on healthy individuals, and provided a comprehensive analysis of the performance of a short-latency brain switch. The morphological investigation showed a cross-subject consistency of a prolonged negative phase in MRCP, and a delayed beta rebound in sensory-motor rhythms during repetitive tasks.

## (B) systems for neuromodulation based on DBS, EMG, and SNN

Electrical stimulation and neuromodulation are techniques used as therapeutic solutions for neurorehabilitation. We distinguish here three approaches: DBS, EMG-based electrical stimulation, and SNN for biomimetic micro-stimulations.

Rossi et al. review the proceedings of the *3rd Annual Deep Brain Stimulation Think Tank* which discussed the most contemporary clinical, electrophysiological, imaging, and computational work on DBS for the treatment of neurological and neuropsychiatric disease. Recent evidence suggests that DBS of the STN in Parkinson's disease mediates its clinical effects by modulating cortical oscillatory activity, presumably via a direct cortico-subthalamic connection. This observation might pave the way for novel closed-loop approaches comprising a cortical sensor. Kern et al. follow the same direction and provide preliminary evidence for detecting a cortical fingerprint of Parkinson's disease for closed-loop neuromodulation.

By following a different approach, Attiah et al. perform closed-loop experiments for reanimating paralyzed facial muscles in a rodent model. The EMG signal of the intact side was used to trigger Functional Electrical Stimulation (FES) on the paralyzed side to restore symmetric whisking. The results demonstrate a novel *in vivo* platform for developing control strategies for neuromuscular facial prostheses. Time-variant muscle responses under ES are often problematic for all the applications of neuroprosthetic muscle control. Hayashibe overviews the background of this topic and highlights important points to be aware of when using ES to induce the desired muscle activation regardless of the time-variance. He also demonstrates how to deal with the common critical problem of ES to move toward robust neuroprosthetic muscle control with the evoked electromyographically controlled electrical stimulation paradigm.

Neural prostheses based on electrical micro-stimulation offer promising perspectives to restore lost functions following lesions of the Central Nervous System (CNS). A challenging perspective is to control micro-stimulation by SNN hybridized to the living tissue. Joucla et al. design an artificial Central Pattern Generator (CPG) based on real-time SNN to generate alternating activity. This system is hybridized to living spinal cord to drive electrical micro-stimulation. These results are a first step toward hybrid artificial/biological solutions based on electrical micro-stimulation for the restoration of lost function in the injured CNS. Spinal cord injury can disrupt connections between the brain respiratory network and the respiratory muscles which can lead to partial or complete loss of ventilatory control and require ventilatory assistance. Zbrzeski et al. present an original bio-inspired technology for real-time ventilation assistance, implemented in a digital circuit. The bio-inspired controller, which is a SNN inspired by the medullary respiratory network, is as robust as a classical controller while exhibiting a flexible, low-power and low-cost hardware design.

## (C) closed-loop systems with BMI for rehabilitation

In this section, we focus on closed-loop systems for rehabilitation. BMIs may support motor impaired patients during activities of daily living by controlling external devices such as prostheses (assistive BMI). Wright et al. review control strategies in existing experimental, investigational and clinical neuroprosthetic systems in order to establish a baseline and promote a common understanding of different feedback modalities and closed-loop controllers.

The closed-loop control of rehabilitative technologies by neural commands has shown a great potential to improve motor recovery in patients suffering from paralysis. BMIs can be used as a natural control method for such technologies. Lopez-Larraz et al. present a proof-of-concept study to validate the feasibility of a BMI to control an ambulatory exoskeleton by patients with incomplete paraplegia. Ferrante et al. design a personalized multi-channel FES controller for gait training, integrating three novel aspects: (1) the FES strategy was based on healthy muscle synergies in order to mimic the neural solutions adopted by the CNS to generate locomotion; (2) the FES strategy was personalized according to an initial locomotion assessment of the patient and was designed to specifically activate the impaired biomechanical functions; (3) the FES strategy was mapped accurately on the altered gait kinematics providing a maximal synchronization between patient's volitional gait and stimulation patterns.

Different studies on rehabilitation of stroke patients are presented in this research topic. (Ibanez et al.; Ibanez et al.) explore the feasibility of using BMIs providing low-latency support to upper-limb reaching movements in patients with stroke as a reliable and usable solution for motor rehabilitation interventions with potential functional benefits.

Stroke patients with severe motor deficits cannot execute task-oriented rehabilitation exercises with their affected upper extremity. Advanced rehabilitation technology may support them in performing such reach-to-grasp movements. The challenge is, however, to provide assistance as needed, while maintaining the participants' commitment during the exercises. In a feasibility study, Grimm and Gharabaghi introduce a closed-loop neuroprosthesis for reach-to-grasp assistance which combines adaptive multi-channel neuromuscular stimulation with a multi-joint arm exoskeleton. Grimm et al. also combine a hybrid BMI with neuromuscular stimulation and antigravity assistance which augments upper limb function and brain activity during rehabilitation exercises and may thus provide a novel restorative framework for severely affected stroke patients. Combining gravity-compensating assistance with adaptive closed-loop feedback in virtual reality provides customized rehabilitation environments for severely affected stroke patients. Grimm et al. develop this approach to simplify motor learning by progressively challenging the subject in accordance with the individual capacity for functional restoration. Bhagat et al. demonstrate the feasibility of detecting motor intent from brain activity of chronic stroke patients using an asynchronous EEG-based BMI. Intent was inferred from movement related cortical potentials (MRCPs) measured over an optimized set of EEG electrodes. These findings provide evidence that closed-loop EEG-based BMI for stroke patients can be designed and optimized to successfully perform across multiple days without system recalibration.

To conclude this part, Gharabaghi wrote a perspective article discussing the necessary features and prerequisites of restorative BMI for stroke rehabilitation.

## Author contributions

TL, PB, PM, and MC: Prepared and discussed about this research topic, invited authors, revised their manuscripts, and handled their revisions.

### Conflict of interest statement

The authors declare that the research was conducted in the absence of any commercial or financial relationships that could be construed as a potential conflict of interest.
